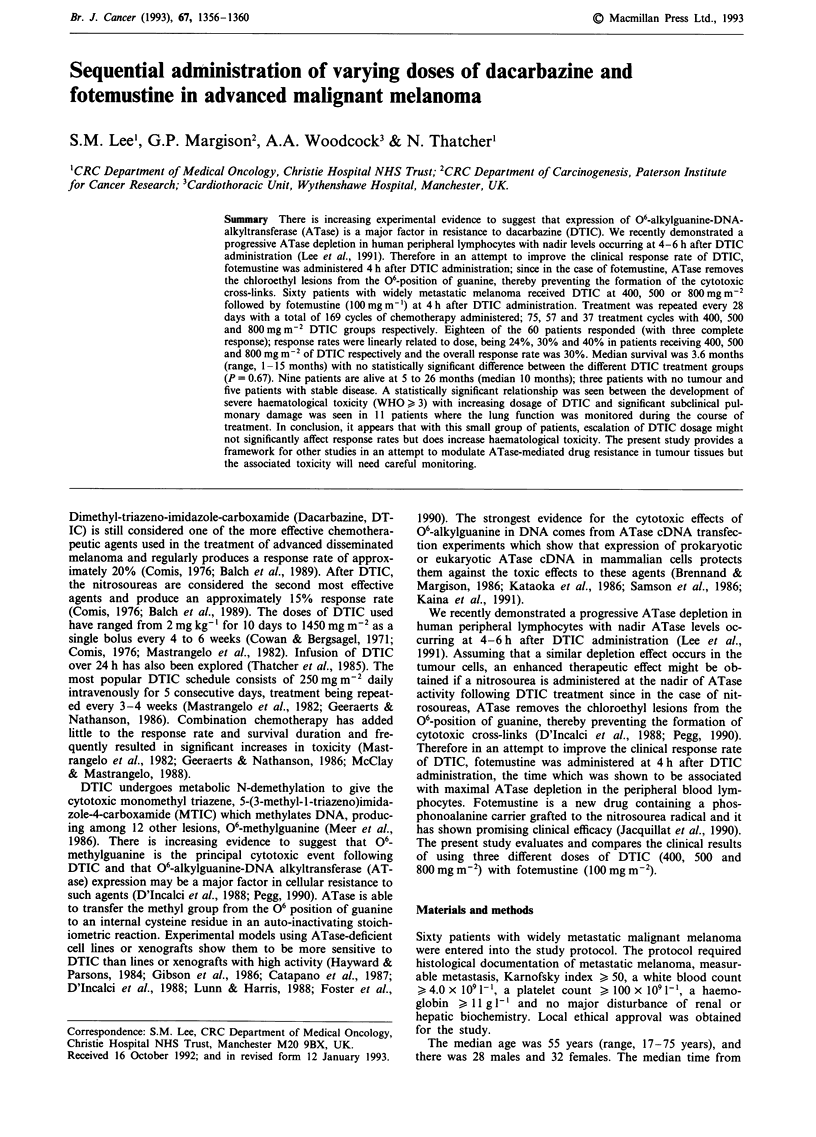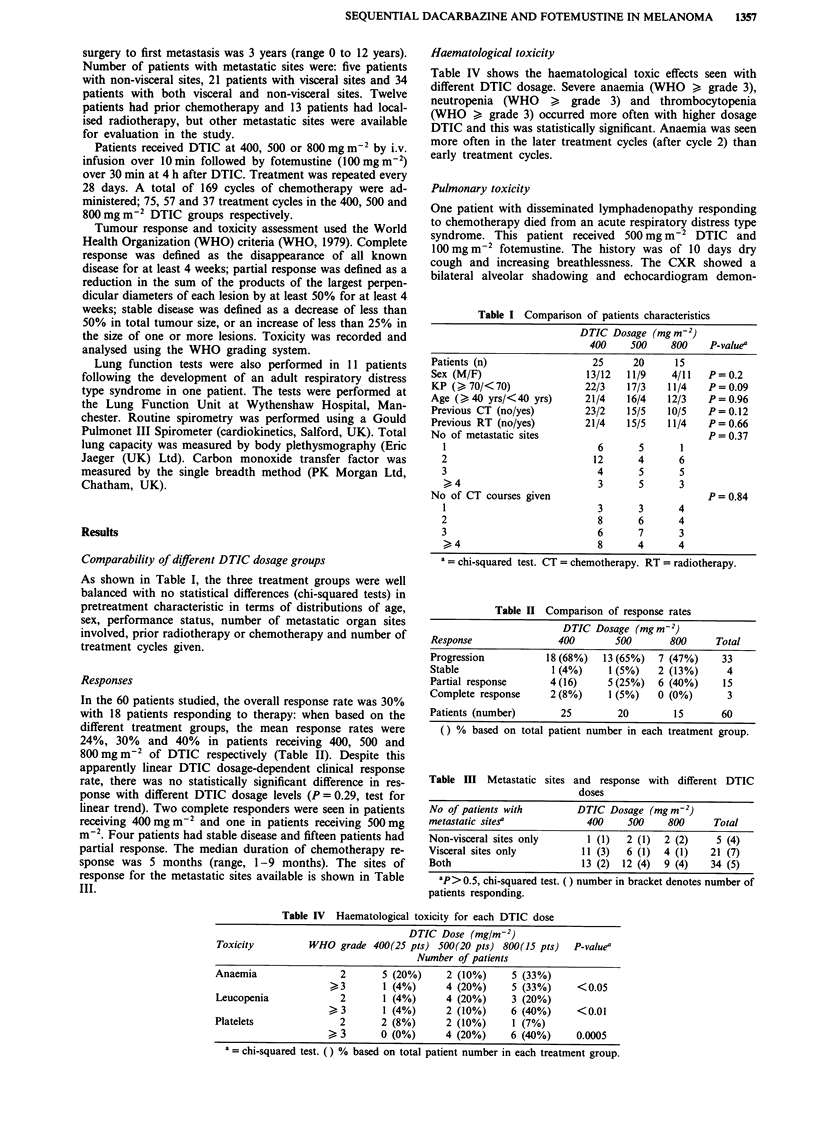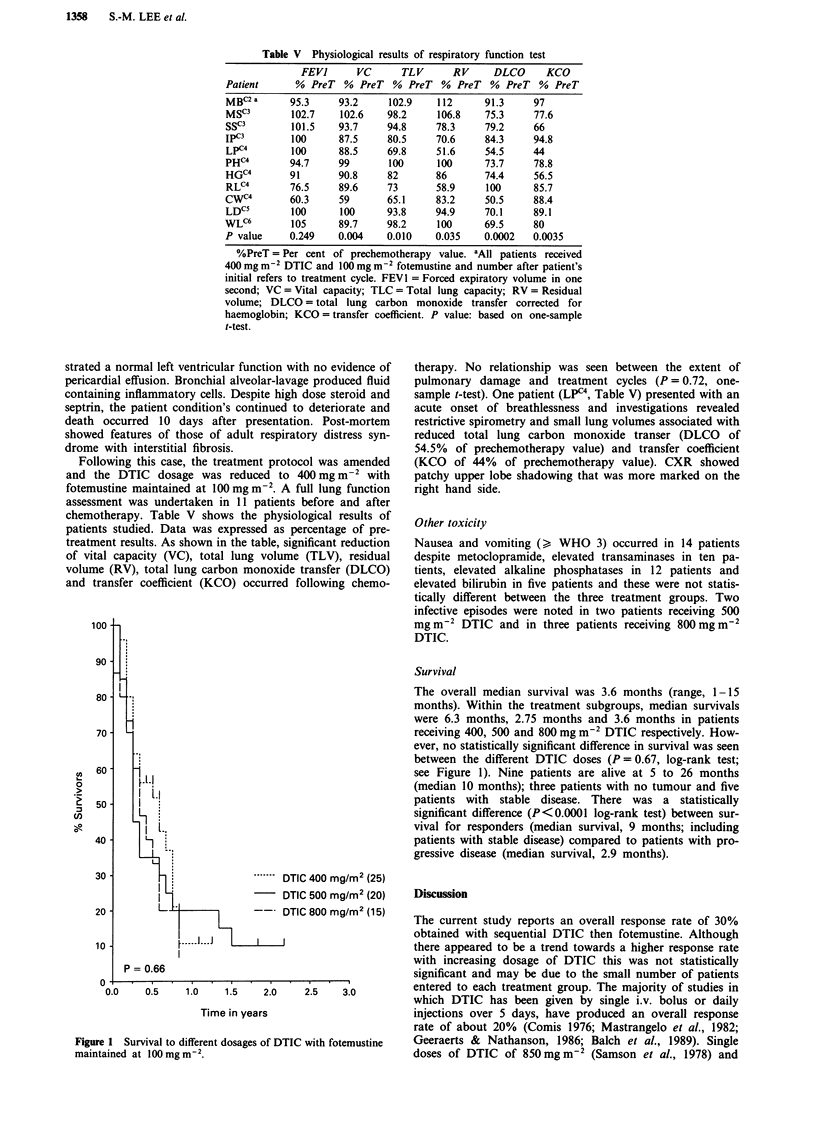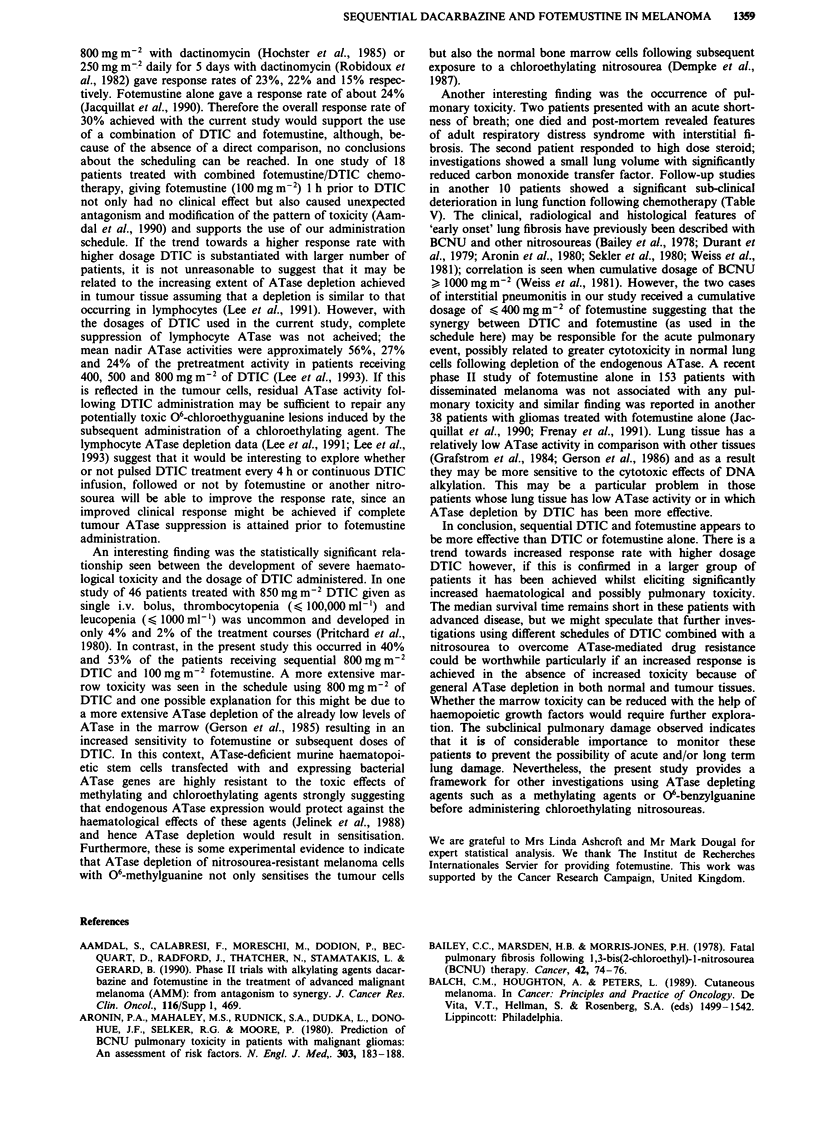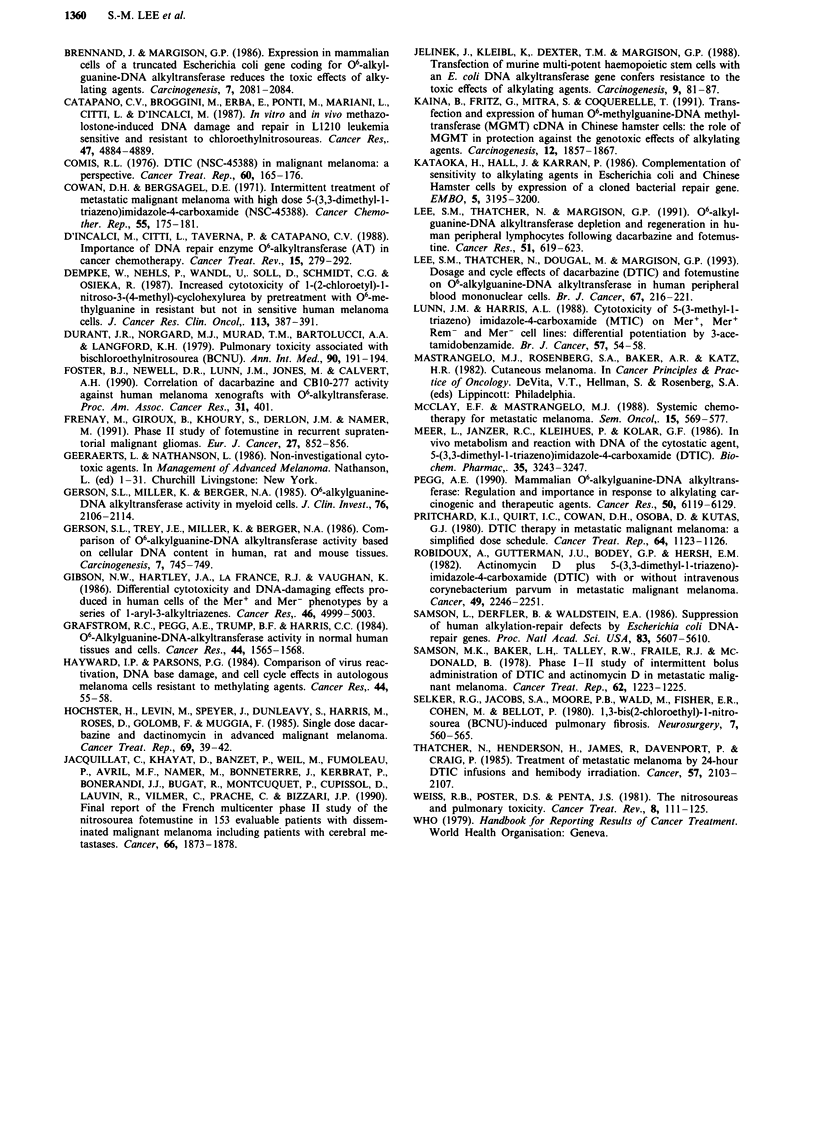# Sequential administration of varying doses of dacarbazine and fotemustine in advanced malignant melanoma.

**DOI:** 10.1038/bjc.1993.251

**Published:** 1993-06

**Authors:** S. M. Lee, G. P. Margison, A. A. Woodcock, N. Thatcher

**Affiliations:** CRC Department of Medical Oncology, Christie Hospital NHS Trust, Manchester, U.K.

## Abstract

There is increasing experimental evidence to suggest that expression of O6-alkylguanine-DNA-alkyltransferase (ATase) is a major factor in resistance to dacarbazine (DTIC). We recently demonstrated a progressive ATase depletion in human peripheral lymphocytes with nadir levels occurring at 4-6 h after DTIC administration (Lee et al., 1991). Therefore in an attempt to improve the clinical response rate of DTIC, fotemustine was administered 4 h after DTIC administration; since in the case of fotemustine, ATase removes the chloroethyl lesions from the O6-position of guanine, thereby preventing the formation of the cytotoxic cross-links. Sixty patients with widely metastatic melanoma received DTIC at 400, 500 or 800 mg m-2 followed by fotemustine (100 mg m-1) at 4 h after DTIC administration. Treatment was repeated every 28 days with a total of 169 cycles of chemotherapy administered; 75, 57 and 37 treatment cycles with 400, 500 and 800 mg m-2 DTIC groups respectively. Eighteen of the 60 patients responded (with three complete response); response rates were linearly related to dose, being 24%, 30% and 40% in patients receiving 400, 500 and 800 mg m-2 of DTIC respectively and the overall response rate was 30%. Median survival was 3.6 months (range, 1-15 months) with no statistically significant difference between the different DTIC treatment groups (P = 0.67). Nine patients are alive at 5 to 26 months (median 10 months); three patients with no tumour and five patients with stable disease. A statistically significant relationship was seen between the development of severe haematological toxicity (WHO > or = 3) with increasing dosage of DTIC and significant subclinical pulmonary damage was seen in 11 patients where the lung function was monitored during the course of treatment. In conclusion, it appears that with this small group of patients, escalation of DTIC dosage might not significantly affect response rates but does increase haematological toxicity. The present study provides a framework for other studies in an attempt to modulate ATase-mediated drug resistance in tumour tissues but the associated toxicity will need careful monitoring.


Br. J. Cancer (1993), 67, 1356-1360 © Macmillan Press Ltd., 1993

### Sequential administration of varying doses of dacarbazine and fotemustine in advanced malignant melanoma

S.M. Lee[1], G.P. Margison[2], A.A. Woodcock[3] & N. Thatcher[1]

[1]*CRC Department of Medical Oncology, Christie Hospital NHS Trust;* [2]*CRC Department of Carcinogenesis, Paterson Institute for Cancer Research;* [3]*Cardiothoracic Unit, Wythenshawe Hospital, Manchester, UK.*

**Summary** There is increasing experimental evidence to suggest that expression of $O^6$-alkylguanine-DNA-alkyltransferase (ATase) is a major factor in resistance to dacarbazine (DTIC). We recently demonstrated a progressive ATase depletion in human peripheral lymphocytes with nadir levels occurring at 4–6 h after DTIC administration (Lee *et al.*, 1991). Therefore in an attempt to improve the clinical response rate of DTIC, fotemustine was administered 4 h after DTIC administration; since in the case of fotemustine, ATase removes the chloroethyl lesions from the $O^6$-position of guanine, thereby preventing the formation of the cytotoxic cross-links. Sixty patients with widely metastatic melanoma received DTIC at 400, 500 or 800 mg m$^{-2}$ followed by fotemustine (100 mg m$^{-1}$) at 4 h after DTIC administration. Treatment was repeated every 28 days with a total of 169 cycles of chemotherapy administered; 75, 57 and 37 treatment cycles with 400, 500 and 800 mg m$^{-2}$ DTIC groups respectively. Eighteen of the 60 patients responded (with three complete response); response rates were linearly related to dose, being 24%, 30% and 40% in patients receiving 400, 500 and 800 mg m$^{-2}$ of DTIC respectively and the overall response rate was 30%. Median survival was 3.6 months (range, 1–15 months) with no statistically significant difference between the different DTIC treatment groups ($P = 0.67$). Nine patients are alive at 5 to 26 months (median 10 months); three patients with no tumour and five patients with stable disease. A statistically significant relationship was seen between the development of severe haematological toxicity (WHO $\geqslant 3$) with increasing dosage of DTIC and significant subclinical pulmonary damage was seen in 11 patients where the lung function was monitored during the course of treatment. In conclusion, it appears that with this small group of patients, escalation of DTIC dosage might not significantly affect response rates but does increase haematological toxicity. The present study provides a framework for other studies in an attempt to modulate ATase-mediated drug resistance in tumour tissues but the associated toxicity will need careful monitoring.

Dimethyl-triazeno-imidazole-carboxamide (Dacarbazine, DTIC) is still considered one of the more effective chemotherapeutic agents used in the treatment of advanced disseminated melanoma and regularly produces a response rate of approximately 20% (Comis, 1976; Balch *et al.*, 1989). After DTIC, the nitrosoureas are considered the second most effective agents and produce an approximately 15% response rate (Comis, 1976; Balch *et al.*, 1989). The doses of DTIC used have ranged from 2 mg kg$^{-1}$ for 10 days to 1450 mg m$^{-2}$ as a single bolus every 4 to 6 weeks (Cowan & Bergsagel, 1971; Comis, 1976; Mastrangelo *et al.*, 1982). Infusion of DTIC over 24 h has also been explored (Thatcher *et al.*, 1985). The most popular DTIC schedule consists of 250 mg m$^{-2}$ daily intravenously for 5 consecutive days, treatment being repeated every 3–4 weeks (Mastrangelo *et al.*, 1982; Geeraerts & Nathanson, 1986). Combination chemotherapy has added little to the response rate and survival duration and frequently resulted in significant increases in toxicity (Mastrangelo *et al.*, 1982; Geeraerts & Nathanson, 1986; McClay & Mastrangelo, 1988).

DTIC undergoes metabolic N-demethylation to give the cytotoxic monomethyl triazene, 5-(3-methyl-1-triazeno)imidazole-4-carboxamide (MTIC) which methylates DNA, producing among 12 other lesions, $O^6$-methylguanine (Meer *et al.*, 1986). There is increasing evidence to suggest that $O^6$-methylguanine is the principal cytotoxic event following DTIC and that $O^6$-alkylguanine-DNA alkyltransferase (ATase) expression may be a major factor in cellular resistance to such agents (D'Incalci *et al.*, 1988; Pegg, 1990). ATase is able to transfer the methyl group from the $O^6$ position of guanine to an internal cysteine residue in an auto-inactivating stoichiometric reaction. Experimental models using ATase-deficient cell lines or xenografts show them to be more sensitive to DTIC than lines or xenografts with high activity (Hayward & Parsons, 1984; Gibson *et al.*, 1986; Catapano *et al.*, 1987; D'Incalci *et al.*, 1988; Lunn & Harris, 1988; Foster *et al.*, 1990). The strongest evidence for the cytotoxic effects of $O^6$-alkylguanine in DNA comes from ATase cDNA transfection experiments which show that expression of prokaryotic or eukaryotic ATase cDNA in mammalian cells protects them against the toxic effects to these agents (Brennand & Margison, 1986; Kataoka *et al.*, 1986; Samson *et al.*, 1986; Kaina *et al.*, 1991).

We recently demonstrated a progressive ATase depletion in human peripheral lymphocytes with nadir ATase levels occurring at 4–6 h after DTIC administration (Lee *et al.*, 1991). Assuming that a similar depletion effect occurs in the tumour cells, an enhanced therapeutic effect might be obtained if a nitrosourea is administered at the nadir of ATase activity following DTIC treatment since in the case of nitrosoureas, ATase removes the chloroethyl lesions from the $O^6$-position of guanine, thereby preventing the formation of cytotoxic cross-links (D'Incalci *et al.*, 1988; Pegg, 1990). Therefore in an attempt to improve the clinical response rate of DTIC, fotemustine was administered at 4 h after DTIC administration, the time which was shown to be associated with maximal ATase depletion in the peripheral blood lymphocytes. Fotemustine is a new drug containing a phosphonoalanine carrier grafted to the nitrosourea radical and it has shown promising clinical efficacy (Jacquillat *et al.*, 1990). The present study evaluates and compares the clinical results of using three different doses of DTIC (400, 500 and 800 mg m$^{-2}$) with fotemustine (100 mg m$^{-2}$).

### Materials and methods

Sixty patients with widely metastatic malignant melanoma were entered into the study protocol. The protocol required histological documentation of metastatic melanoma, measurable metastasis, Karnofsky index $\geqslant 50$, a white blood count $\geqslant 4.0 \times 10^9 \, l^{-1}$, a platelet count $\geqslant 100 \times 10^9 \, l^{-1}$, a haemoglobin $\geqslant 11 \, g \, l^{-1}$ and no major disturbance of renal or hepatic biochemistry. Local ethical approval was obtained for the study.

The median age was 55 years (range, 17–75 years), and there was 28 males and 32 females. The median time from

Correspondence: S.M. Lee, CRC Department of Medical Oncology, Christie Hospital NHS Trust, Manchester M20 9BX, UK.
Received 16 October 1992; and in revised form 12 January 1993.

surgery to first metastasis was 3 years (range 0 to 12 years). Number of patients with metastatic sites were: five patients with non-visceral sites, 21 patients with visceral sites and 34 patients with both visceral and non-visceral sites. Twelve patients had prior chemotherapy and 13 patients had localised radiotherapy, but other metastatic sites were available for evaluation in the study.

Patients received DTIC at 400, 500 or 800 mg $m^{-2}$ by i.v. infusion over 10 min followed by fotemustine (100 mg $m^{-2}$) over 30 min at 4 h after DTIC. Treatment was repeated every 28 days. A total of 169 cycles of chemotherapy were administered; 75, 57 and 37 treatment cycles in the 400, 500 and 800 mg $m^{-2}$ DTIC groups respectively.

Tumour response and toxicity assessment used the World Health Organization (WHO) criteria (WHO, 1979). Complete response was defined as the disappearance of all known disease for at least 4 weeks; partial response was defined as a reduction in the sum of the products of the largest perpendicular diameters of each lesion by at least 50% for at least 4 weeks; stable disease was defined as a decrease of less than 50% in total tumour size, or an increase of less than 25% in the size of one or more lesions. Toxicity was recorded and analysed using the WHO grading system.

Lung function tests were also performed in 11 patients following the development of an adult respiratory distress type syndrome in one patient. The tests were performed at the Lung Function Unit at Wythenshaw Hospital, Manchester. Routine spirometry was performed using a Gould Pulmonet III Spirometer (cardiokinetics, Salford, UK). Total lung capacity was measured by body plethysmography (Eric Jaeger (UK) Ltd). Carbon monoxide transfer factor was measured by the single breadth method (PK Morgan Ltd, Chatham, UK).

### Results

*Comparability of different DTIC dosage groups*

As shown in Table I, the three treatment groups were well balanced with no statistical differences (chi-squared tests) in pretreatment characteristic in terms of distributions of age, sex, performance status, number of metastatic organ sites involved, prior radiotherapy or chemotherapy and number of treatment cycles given.

*Responses*

In the 60 patients studied, the overall response rate was 30% with 18 patients responding to therapy: when based on the different treatment groups, the mean response rates were 24%, 30% and 40% in patients receiving 400, 500 and 800 mg $m^{-2}$ of DTIC respectively (Table II). Despite this apparently linear DTIC dosage-dependent clinical response rate, there was no statistically significant difference in response with different DTIC dosage levels ($P = 0.29$, test for linear trend). Two complete responders were seen in patients receiving 400 mg $m^{-2}$ and one in patients receiving 500 mg $m^{-2}$. Four patients had stable disease and fifteen patients had partial response. The median duration of chemotherapy response was 5 months (range, 1–9 months). The sites of response for the metastatic sites available is shown in Table III.

*Haematological toxicity*

Table IV shows the haematological toxic effects seen with different DTIC dosage. Severe anaemia (WHO ⩾ grade 3), neutropenia (WHO ⩾ grade 3) and thrombocytopenia (WHO ⩾ grade 3) occurred more often with higher dosage DTIC and this was statistically significant. Anaemia was seen more often in the later treatment cycles (after cycle 2) than early treatment cycles.

*Pulmonary toxicity*

One patient with disseminated lymphadenopathy responding to chemotherapy died from an acute respiratory distress type syndrome. This patient received 500 mg $m^{-2}$ DTIC and 100 mg $m^{-2}$ fotemustine. The history was of 10 days dry cough and increasing breathlessness. The CXR showed a bilateral alveolar shadowing and echocardiogram demon-

**Table I** Comparison of patients characteristics

| | *DTIC Dosage (mg m⁻²)* 400 | 500 | 800 | *P-value*[a] |
|---|---|---|---|---|
| Patients (n) | 25 | 20 | 15 | |
| Sex (M/F) | 13/12 | 11/9 | 4/11 | $P = 0.2$ |
| KP (⩾ 70/< 70) | 22/3 | 17/3 | 11/4 | $P = 0.09$ |
| Age (⩾ 40 yrs/< 40 yrs) | 21/4 | 16/4 | 12/3 | $P = 0.96$ |
| Previous CT (no/yes) | 23/2 | 15/5 | 10/5 | $P = 0.12$ |
| Previous RT (no/yes) | 21/4 | 15/5 | 11/4 | $P = 0.66$ |
| No of metastatic sites | | | | $P = 0.37$ |
| 1 | 6 | 5 | 1 | |
| 2 | 12 | 4 | 6 | |
| 3 | 4 | 5 | 5 | |
| ⩾ 4 | 3 | 5 | 3 | |
| No of CT courses given | | | | $P = 0.84$ |
| 1 | 3 | 3 | 4 | |
| 2 | 8 | 6 | 4 | |
| 3 | 6 | 7 | 3 | |
| ⩾ 4 | 8 | 4 | 4 | |

[a] = chi-squared test. CT = chemotherapy. RT = radiotherapy.

**Table II** Comparison of response rates

| *Response* | *DTIC Dosage (mg m⁻²)* 400 | 500 | 800 | *Total* |
|---|---|---|---|---|
| Progression | 18 (68%) | 13 (65%) | 7 (47%) | 33 |
| Stable | 1 (4%) | 1 (5%) | 2 (13%) | 4 |
| Partial response | 4 (16) | 5 (25%) | 6 (40%) | 15 |
| Complete response | 2 (8%) | 1 (5%) | 0 (0%) | 3 |
| Patients (number) | 25 | 20 | 15 | 60 |

( ) % based on total patient number in each treatment group.

**Table III** Metastatic sites and response with different DTIC doses

| *No of patients with metastatic sites*[a] | *DTIC Dosage (mg m⁻²)* 400 | 500 | 800 | *Total* |
|---|---|---|---|---|
| Non-visceral sites only | 1 (1) | 2 (1) | 2 (2) | 5 (4) |
| Visceral sites only | 11 (3) | 6 (1) | 4 (1) | 21 (7) |
| Both | 13 (2) | 12 (4) | 9 (4) | 34 (5) |

[a]$P > 0.5$, chi-squared test. ( ) number in bracket denotes number of patients responding.

**Table IV** Haematological toxicity for each DTIC dose

| *Toxicity* | *WHO grade* | *DTIC Dose (mg/m⁻²)* 400(25 pts) | 500(20 pts) | 800(15 pts) *Number of patients* | *P-value*[a] |
|---|---|---|---|---|---|
| Anaemia | 2 | 5 (20%) | 2 (10%) | 5 (33%) | |
| | ⩾ 3 | 1 (4%) | 4 (20%) | 5 (33%) | <0.05 |
| Leucopenia | 2 | 1 (4%) | 4 (20%) | 3 (20%) | |
| | ⩾ 3 | 1 (4%) | 2 (10%) | 6 (40%) | <0.01 |
| Platelets | 2 | 2 (8%) | 2 (10%) | 1 (7%) | |
| | ⩾ 3 | 0 (0%) | 4 (20%) | 6 (40%) | 0.0005 |

[a] = chi-squared test. ( ) % based on total patient number in each treatment group.

**Table V** Physiological results of respiratory function test

| Patient | FEV1 % PreT | VC % PreT | TLV % PreT | RV % PreT | DLCO % PreT | KCO % PreT |
|---|---|---|---|---|---|---|
| MB[C2 a] | 95.3 | 93.2 | 102.9 | 112 | 91.3 | 97 |
| MS[C3] | 102.7 | 102.6 | 98.2 | 106.8 | 75.3 | 77.6 |
| SS[C3] | 101.5 | 93.7 | 94.8 | 78.3 | 79.2 | 66 |
| IP[C3] | 100 | 87.5 | 80.5 | 70.6 | 84.3 | 94.8 |
| LP[C4] | 100 | 88.5 | 69.8 | 51.6 | 54.5 | 44 |
| PH[C4] | 94.7 | 99 | 100 | 100 | 73.7 | 78.8 |
| HG[C4] | 91 | 90.8 | 82 | 86 | 74.4 | 56.5 |
| RL[C4] | 76.5 | 89.6 | 73 | 58.9 | 100 | 85.7 |
| CW[C4] | 60.3 | 59 | 65.1 | 83.2 | 50.5 | 88.4 |
| LD[C5] | 100 | 100 | 93.8 | 94.9 | 70.1 | 89.1 |
| WL[C6] | 105 | 89.7 | 98.2 | 100 | 69.5 | 80 |
| *P* value | 0.249 | 0.004 | 0.010 | 0.035 | 0.0002 | 0.0035 |

%PreT = Per cent of prechemotherapy value. [a]All patients received 400 mg m$^{-2}$ DTIC and 100 mg m$^{-2}$ fotemustine and number after patient's initial refers to treatment cycle. FEV1 = Forced expiratory volume in one second; VC = Vital capacity; TLC = Total lung capacity; RV = Residual volume; DLCO = total lung carbon monoxide transfer corrected for haemoglobin; KCO = transfer coefficient. *P* value: based on one-sample *t*-test.

strated a normal left ventricular function with no evidence of pericardial effusion. Bronchial alveolar-lavage produced fluid containing inflammatory cells. Despite high dose steroid and septrin, the patient condition's continued to deteriorate and death occurred 10 days after presentation. Post-mortem showed features of those of adult respiratory distress syndrome with interstitial fibrosis.

Following this case, the treatment protocol was amended and the DTIC dosage was reduced to 400 mg m$^{-2}$ with fotemustine maintained at 100 mg m$^{-2}$. A full lung function assessment was undertaken in 11 patients before and after chemotherapy. Table V shows the physiological results of patients studied. Data was expressed as percentage of pre-treatment results. As shown in the table, significant reduction of vital capacity (VC), total lung volume (TLV), residual volume (RV), total lung carbon monoxide transfer (DLCO) and transfer coefficient (KCO) occurred following chemotherapy. No relationship was seen between the extent of pulmonary damage and treatment cycles ($P = 0.72$, one-sample *t*-test). One patient (LP[C4], Table V) presented with an acute onset of breathlessness and investigations revealed restrictive spirometry and small lung volumes associated with reduced total lung carbon monoxide transer (DLCO of 54.5% of prechemotherapy value) and transfer coefficient (KCO of 44% of prechemotherapy value). CXR showed patchy upper lobe shadowing that was more marked on the right hand side.

**Figure 1** Survival to different dosages of DTIC with fotemustine maintained at 100 mg m$^{-2}$.

*Other toxicity*

Nausea and vomiting ($\geqslant$ WHO 3) occurred in 14 patients despite metoclopramide, elevated transaminases in ten patients, elevated alkaline phosphatases in 12 patients and elevated bilirubin in five patients and these were not statistically different between the three treatment groups. Two infective episodes were noted in two patients receiving 500 mg m$^{-2}$ DTIC and in three patients receiving 800 mg m$^{-2}$ DTIC.

*Survival*

The overall median survival was 3.6 months (range, 1-15 months). Within the treatment subgroups, median survivals were 6.3 months, 2.75 months and 3.6 months in patients receiving 400, 500 and 800 mg m$^{-2}$ DTIC respectively. However, no statistically significant difference in survival was seen between the different DTIC doses ($P = 0.67$, log-rank test; see Figure 1). Nine patients are alive at 5 to 26 months (median 10 months); three patients with no tumour and five patients with stable disease. There was a statistically significant difference ($P < 0.0001$ log-rank test) between survival for responders (median survival, 9 months; including patients with stable disease) compared to patients with progressive disease (median survival, 2.9 months).

### Discussion

The current study reports an overall response rate of 30% obtained with sequential DTIC then fotemustine. Although there appeared to be a trend towards a higher response rate with increasing dosage of DTIC this was not statistically significant and may be due to the small number of patients entered to each treatment group. The majority of studies in which DTIC has been given by single i.v. bolus or daily injections over 5 days, have produced an overall response rate of about 20% (Comis 1976; Mastrangelo *et al.*, 1982; Geeraerts & Nathanson, 1986; Balch *et al.*, 1989). Single doses of DTIC of 850 mg m$^{-2}$ (Samson *et al.*, 1978) and

800 mg m$^{-2}$ with dactinomycin (Hochster *et al.*, 1985) or 250 mg m$^{-2}$ daily for 5 days with dactinomycin (Robidoux *et al.*, 1982) gave response rates of 23%, 22% and 15% respectively. Fotemustine alone gave a response rate of about 24% (Jacquillat *et al.*, 1990). Therefore the overall response rate of 30% achieved with the current study would support the use of a combination of DTIC and fotemustine, although, because of the absence of a direct comparison, no conclusions about the scheduling can be reached. In one study of 18 patients treated with combined fotemustine/DTIC chemotherapy, giving fotemustine (100 mg m$^{-2}$) 1 h prior to DTIC not only had no clinical effect but also caused unexpected antagonism and modification of the pattern of toxicity (Aamdal *et al.*, 1990) and supports the use of our administration schedule. If the trend towards a higher response rate with higher dosage DTIC is substantiated with larger number of patients, it is not unreasonable to suggest that it may be related to the increasing extent of ATase depletion achieved in tumour tissue assuming that a depletion is similar to that occurring in lymphocytes (Lee *et al.*, 1991). However, with the dosages of DTIC used in the current study, complete suppression of lymphocyte ATase was not acheived; the mean nadir ATase activities were approximately 56%, 27% and 24% of the pretreatment activity in patients receiving 400, 500 and 800 mg m$^{-2}$ of DTIC (Lee *et al.*, 1993). If this is reflected in the tumour cells, residual ATase activity following DTIC administration may be sufficient to repair any potentially toxic O$^6$-chloroethyguanine lesions induced by the subsequent administration of a chloroethylating agent. The lymphocyte ATase depletion data (Lee *et al.*, 1991; Lee *et al.*, 1993) suggest that it would be interesting to explore whether or not pulsed DTIC treatment every 4 h or continuous DTIC infusion, followed or not by fotemustine or another nitrosourea will be able to improve the response rate, since an improved clinical response might be achieved if complete tumour ATase suppression is attained prior to fotemustine administration.

An interesting finding was the statistically significant relationship seen between the development of severe haematological toxicity and the dosage of DTIC administered. In one study of 46 patients treated with 850 mg m$^{-2}$ DTIC given as single i.v. bolus, thrombocytopenia ($\leqslant$ 100,000 ml$^{-1}$) and leucopenia ($\leqslant$ 1000 ml$^{-1}$) was uncommon and developed in only 4% and 2% of the treatment courses (Pritchard *et al.*, 1980). In contrast, in the present study this occurred in 40% and 53% of the patients receiving sequential 800 mg m$^{-2}$ DTIC and 100 mg m$^{-2}$ fotemustine. A more extensive marrow toxicity was seen in the schedule using 800 mg m$^{-2}$ of DTIC and one possible explanation for this might be due to a more extensive ATase depletion of the already low levels of ATase in the marrow (Gerson *et al.*, 1985) resulting in an increased sensitivity to fotemustine or subsequent doses of DTIC. In this context, ATase-deficient murine haematopoietic stem cells transfected with and expressing bacterial ATase genes are highly resistant to the toxic effects of methylating and chloroethylating agents strongly suggesting that endogenous ATase expression would protect against the haematological effects of these agents (Jelinek *et al.*, 1988) and hence ATase depletion would result in sensitisation. Furthermore, these is some experimental evidence to indicate that ATase depletion of nitrosourea-resistant melanoma cells with O$^6$-methylguanine not only sensitises the tumour cells but also the normal bone marrow cells following subsequent exposure to a chloroethylating nitrosourea (Dempke *et al.*, 1987).

Another interesting finding was the occurrence of pulmonary toxicity. Two patients presented with an acute shortness of breath; one died and post-mortem revealed features of adult respiratory distress syndrome with interstitial fibrosis. The second patient responded to high dose steroid; investigations showed a small lung volume with significantly reduced carbon monoxide transfer factor. Follow-up studies in another 10 patients showed a significant sub-clinical deterioration in lung function following chemotherapy (Table V). The clinical, radiological and histological features of 'early onset' lung fibrosis have previously been described with BCNU and other nitrosoureas (Bailey *et al.*, 1978; Durant *et al.*, 1979; Aronin *et al.*, 1980; Sekler *et al.*, 1980; Weiss *et al.*, 1981); correlation is seen when cumulative dosage of BCNU $\geqslant$ 1000 mg m$^{-2}$ (Weiss *et al.*, 1981). However, the two cases of interstitial pneumonitis in our study received a cumulative dosage of $\leqslant$ 400 mg m$^{-2}$ of fotemustine suggesting that the synergy between DTIC and fotemustine (as used in the schedule here) may be responsible for the acute pulmonary event, possibly related to greater cytotoxicity in normal lung cells following depletion of the endogenous ATase. A recent phase II study of fotemustine alone in 153 patients with disseminated melanoma was not associated with any pulmonary toxicity and similar finding was reported in another 38 patients with gliomas treated with fotemustine alone (Jacquillat *et al.*, 1990; Frenay *et al.*, 1991). Lung tissue has a relatively low ATase activity in comparison with other tissues (Grafstrom *et al.*, 1984; Gerson *et al.*, 1986) and as a result they may be more sensitive to the cytotoxic effects of DNA alkylation. This may be a particular problem in those patients whose lung tissue has low ATase activity or in which ATase depletion by DTIC has been more effective.

In conclusion, sequential DTIC and fotemustine appears to be more effective than DTIC or fotemustine alone. There is a trend towards increased response rate with higher dosage DTIC however, if this is confirmed in a larger group of patients it has been achieved whilst eliciting significantly increased haematological and possibly pulmonary toxicity. The median survival time remains short in these patients with advanced disease, but we might speculate that further investigations using different schedules of DTIC combined with a nitrosourea to overcome ATase-mediated drug resistance could be worthwhile particularly if an increased response is achieved in the absence of increased toxicity because of general ATase depletion in both normal and tumour tissues. Whether the marrow toxicity can be reduced with the help of haemopoietic growth factors would require further exploration. The subclinical pulmonary damage observed indicates that it is of considerable importance to monitor these patients to prevent the possibility of acute and/or long term lung damage. Nevertheless, the present study provides a framework for other investigations using ATase depleting agents such as a methylating agents or O$^6$-benzylguanine before administering chloroethylating nitrosoureas.

We are grateful to Mrs Linda Ashcroft and Mr Mark Dougal for expert statistical analysis. We thank The Institut de Recherches Internationales Servier for providing fotemustine. This work was supported by the Cancer Research Campaign, United Kingdom.

### References

AAMDAL, S., CALABRESI, F., MORESCHI, M., DODION, P., BECQUART, D., RADFORD, J., THATCHER, N., STAMATAKIS, L. & GERARD, B. (1990). Phase II trials with alkylating agents dacarbazine and fotemustine in the treatment of advanced malignant melanoma (AMM): from antagonism to synergy. *J. Cancer Res. Clin. Oncol.*, **116**/Supp 1, 469.

ARONIN, P.A., MAHALEY, M.S., RUDNICK, S.A., DUDKA, L., DONOHUE, J.F., SELKER, R.G. & MOORE, P. (1980). Prediction of BCNU pulmonary toxicity in patients with malignant gliomas: An assessment of risk factors. *N. Engl. J. Med,*. **303**, 183–188.

BAILEY, C.C., MARSDEN, H.B. & MORRIS-JONES, P.H. (1978). Fatal pulmonary fibrosis following 1,3-bis(2-chloroethyl)-1-nitrosourea (BCNU) therapy. *Cancer*, **42**, 74–76.

BALCH, C.M., HOUGHTON, A. & PETERS, L. (1989). Cutaneous melanoma. In *Cancer: Principles and Practice of Oncology*. De Vita, V.T., Hellman, S. & Rosenberg, S.A. (eds) 1499–1542. Lippincott: Philadelphia.

BRENNAND, J. & MARGISON, G.P. (1986). Expression in mammalian cells of a truncated Escherichia coli gene coding for $O^6$-alkylguanine-DNA alkyltransferase reduces the toxic effects of alkylating agents. *Carcinogenesis*, **7**, 2081–2084.

CATAPANO, C.V., BROGGINI, M., ERBA, E., PONTI, M., MARIANI, L., CITTI, L. & D'INCALCI, M. (1987). *In vitro* and *in vivo* methazolostone-induced DNA damage and repair in L1210 leukemia sensitive and resistant to chloroethylnitrosoureas. *Cancer Res*,. **47**, 4884–4889.

COMIS, R.L. (1976). DTIC (NSC-45388) in malignant melanoma: a perspective. *Cancer Treat. Rep.*, **60**, 165–176.

COWAN, D.H. & BERGSAGEL, D.E. (1971). Intermittent treatment of metastatic malignant melanoma with high dose 5-(3,3-dimethyl-1-triazeno)imidazole-4-carboxamide (NSC-45388). *Cancer Chemother. Rep.*, **55**, 175–181.

D'INCALCI, M., CITTI, L., TAVERNA, P. & CATAPANO, C.V. (1988). Importance of DNA repair enzyme $O^6$-alkyltransferase (AT) in cancer chemotherapy. *Cancer Treat. Rev.*, **15**, 279–292.

DEMPKE, W., NEHLS, P., WANDL, U,. SOLL, D., SCHMIDT, C.G. & OSIEKA, R. (1987). Increased cytotoxicity of 1-(2-chloroetyl)-1-nitroso-3-(4-methyl)-cyclohexylurea by pretreatment with $O^6$-methylguanine in resistant but not in sensitive human melanoma cells. *J. Cancer Res. Clin. Oncol*,. **113**, 387–391.

DURANT, J.R., NORGARD, M.J., MURAD, T.M., BARTOLUCCI, A.A. & LANGFORD, K.H. (1979). Pulmonary toxicity associated with bischloroethylnitrosourea (BCNU). *Ann. Int. Med.*, **90**, 191–194.

FOSTER, B.J., NEWELL, D.R., LUNN, J.M., JONES, M. & CALVERT, A.H. (1990). Correlation of dacarbazine and CB10-277 activity against human melanoma xenografts with $O^6$-alkyltransferase. *Proc. Am. Assoc. Cancer Res.*, **31**, 401.

FRENAY, M., GIROUX, B., KHOURY, S., DERLON, J.M. & NAMER, M. (1991). Phase II study of fotemustine in recurrent supratentorial malignant gliomas. *Eur. J. Cancer*, **27**, 852–856.

GEERAERTS, L. & NATHANSON, L. (1986). Non-investigational cytotoxic agents. In *Management of Advanced Melanoma*. Nathanson, L. (ed) 1–31. Churchill Livingstone: New York.

GERSON, S.L., MILLER, K. & BERGER, N.A. (1985). $O^6$-alkylguanine-DNA alkyltransferase activity in myeloid cells. *J. Clin. Invest.*, **76**, 2106–2114.

GERSON, S.L., TREY, J.E., MILLER, K. & BERGER, N.A. (1986). Comparison of $O^6$-alkylguanine-DNA alkyltransferase activity based on cellular DNA content in human, rat and mouse tissues. *Carcinogenesis*, **7**, 745–749.

GIBSON, N.W., HARTLEY, J.A., LA FRANCE, R.J. & VAUGHAN, K. (1986). Differential cytotoxicity and DNA-damaging effects produced in human cells of the $Mer^+$ and $Mer^-$ phenotypes by a series of 1-aryl-3-alkyltriazenes. *Cancer Res*,. **46**, 4999–5003.

GRAFSTROM, R.C., PEGG, A.E., TRUMP, B.F. & HARRIS, C.C. (1984). $O^6$-Alkylguanine-DNA-alkyltransferase activity in normal human tissues and cells. *Cancer Res.*, **44**, 1565–1568.

HAYWARD, I.P. & PARSONS, P.G. (1984). Comparison of virus reactivation, DNA base damage, and cell cycle effects in autologous melanoma cells resistant to methylating agents. *Cancer Res*,. **44**, 55–58.

HOCHSTER, H., LEVIN, M., SPEYER, J., DUNLEAVY, S., HARRIS, M., ROSES, D., GOLOMB, F. & MUGGIA, F. (1985). Single dose dacarbazine and dactinomycin in advanced malignant melanoma. *Cancer Treat. Rep.*, **69**, 39–42.

JACQUILLAT, C., KHAYAT, D., BANZET, P., WEIL, M., FUMOLEAU, P., AVRIL, M.F., NAMER, M., BONNETERRE, J., KERBRAT, P., BONERANDI, J.J., BUGAT, R., MONTCUQUET, P., CUPISSOL, D., LAUVIN, R., VILMER, C., PRACHE, C. & BIZZARI, J.P. (1990). Final report of the French multicenter phase II study of the nitrosourea fotemustine in 153 evaluable patients with disseminated malignant melanoma including patients with cerebral metastases. *Cancer*, **66**, 1873–1878.

JELINEK, J., KLEIBL, K,. DEXTER, T.M. & MARGISON, G.P. (1988). Transfection of murine multi-potent haemopoietic stem cells with an *E. coli* DNA alkyltransferase gene confers resistance to the toxic effects of alkylating agents. *Carcinogenesis*, **9**, 81–87.

KAINA, B., FRITZ, G., MITRA, S. & COQUERELLE, T. (1991). Transfection and expression of human $O^6$-methylguanine-DNA methyltransferase (MGMT) cDNA in Chinese hamster cells: the role of MGMT in protection against the genotoxic effects of alkylating agents. *Carcinogenesis*, **12**, 1857–1867.

KATAOKA, H., HALL, J. & KARRAN, P. (1986). Complementation of sensitivity to alkylating agents in Escherichia coli and Chinese Hamster cells by expression of a cloned bacterial repair gene. *EMBO*, **5**, 3195–3200.

LEE, S.M., THATCHER, N. & MARGISON, G.P. (1991). $O^6$-alkylguanine-DNA alkyltransferase depletion and regeneration in human peripheral lymphocytes following dacarbazine and fotemustine. *Cancer Res.*, **51**, 619–623.

LEE, S.M., THATCHER, N., DOUGAL, M. & MARGISON, G.P. (1993). Dosage and cycle effects of dacarbazine (DTIC) and fotemustine on $O^6$-alkylguanine-DNA alkyltransferase in human peripheral blood mononuclear cells. *Br. J. Cancer*, **67**, 216–221.

LUNN, J.M. & HARRIS, A.L. (1988). Cytotoxicity of 5-(3-methyl-1-triazeno) imidazole-4-carboxamide (MTIC) on $Mer^+$, $Mer^+$ $Rem^-$ and $Mer^-$ cell lines: differential potentiation by 3-acetamidobenzamide. *Br. J. Cancer*, **57**, 54–58.

MASTRANGELO, M.J., ROSENBERG, S.A., BAKER, A.R. & KATZ, H.R. (1982). Cutaneous melanoma. In *Cancer Principles & Practice of Oncology*. DeVita, V.T., Hellman, S. & Rosenberg, S.A. (eds) Lippincott: Philadelphia.

MCCLAY, E.F. & MASTRANGELO, M.J. (1988). Systemic chemotherapy for metastatic melanoma. *Sem. Oncol*,. **15**, 569–577.

MEER, L., JANZER, R.C., KLEIHUES, P. & KOLAR, G.F. (1986). In vivo metabolism and reaction with DNA of the cytostatic agent, 5-(3,3-dimethyl-1-triazeno)imidazole-4-carboxamide (DTIC). *Biochem. Pharmac*,. **35**, 3243–3247.

PEGG, A.E. (1990). Mammalian $O^6$-alkylguanine-DNA alkyltransferase: Regulation and importance in response to alkylating carcinogenic and therapeutic agents. *Cancer Res.*, **50**, 6119–6129.

PRITCHARD, K.I., QUIRT, I.C., COWAN, D.H., OSOBA, D. & KUTAS, G.J. (1980). DTIC therapy in metastatic malignant melanoma: a simplified dose schedule. *Cancer Treat. Rep.*, **64**, 1123–1126.

ROBIDOUX, A., GUTTERMAN, J.U., BODEY, G.P. & HERSH, E.M. (1982). Actinomycin D plus 5-(3,3-dimethyl-1-triazeno)-imidazole-4-carboxamide (DTIC) with or without intravenous corynebacterium parvum in metastatic malignant melanoma. *Cancer*, **49**, 2246–2251.

SAMSON, L., DERFLER, B. & WALDSTEIN, E.A. (1986). Suppression of human alkylation-repair defects by *Escherichia coli* DNA-repair genes. *Proc. Natl Acad. Sci. USA*, **83**, 5607–5610.

SAMSON, M.K., BAKER, L.H,. TALLEY, R.W., FRAILE, R.J. & MCDONALD, B. (1978). Phase I-II study of intermittent bolus administration of DTIC and actinomycin D in metastatic malignant melanoma. *Cancer Treat. Rep.*, **62**, 1223–1225.

SELKER, R.G., JACOBS, S.A., MOORE, P.B., WALD, M., FISHER, E.R., COHEN, M. & BELLOT, P. (1980). 1,3-bis(2-chloroethyl)-1-nitrosourea (BCNU)-induced pulmonary fibrosis. *Neurosurgery*, **7**, 560–565.

THATCHER, N., HENDERSON, H., JAMES, R, DAVENPORT, P. & CRAIG, P. (1985). Treatment of metastatic melanoma by 24-hour DTIC infusions and hemibody irradiation. *Cancer*, **57**, 2103–2107.

WEISS, R.B., POSTER, D.S. & PENTA, J.S. (1981). The nitrosoureas and pulmonary toxicity. *Cancer Treat. Rev.*, **8**, 111–125.

WHO (1979). *Handbook for Reporting Results of Cancer Treatment*. World Health Organisation: Geneva.